# An instrumented approach to quantify wrist and finger flexor spasticity: A study protocol

**DOI:** 10.1371/journal.pone.0328528

**Published:** 2025-07-31

**Authors:** Anna Pennekamp, Ursula Trinler, Julia Janine Glaser, Mirjam Thielen

**Affiliations:** 1 BG Klinik Ludwigshafen, Center for Motion Analysis, Ludwigshafen, Germany; 2 Heidelberg University, Medical Faculty Heidelberg, Heidelberg, Germany; 3 BG Klinik Ludwigshafen, Department of Hand, Plastic and Reconstructive Surgery, Burn Center Plastic and Hand Surgery, University of Heidelberg, Ludwig-Guttmann-Str. 13, Ludwigshafen am Rhein, Germany; 4 BG Klinikum Unfallkrankenhaus Berlin, Department of Hand, Replantation, and Microsurgery and Chair of Hand, Replantation, and Microsurgery at the Charité University Medicine Berlin,; 5 Klinikum St. Elisabeth Straubing, Department of Orthopedic, Trauma and Hand Surgery, St.-Elisabeth-Str. 23, Straubing, Germany; Polytechnic University of Marche: Universita Politecnica delle Marche, ITALY

## Abstract

Spasticity in the upper limb is a common condition observed in individuals with Cerebral Palsy, post-stroke or following traumatic brain injury. Affected patients present with significant functional and care challenges. Advances in both conservative and surgical treatments necessitate improved assessment tools. This study aims to develop and validate an instrumented measurement procedure combining 3D motion analysis, surface electromyography (sEMG), and musculoskeletal modeling to assess wrist and finger spasticity and function. The goal is to create a preoperative assessment tool for surgical strategy determination and a postoperative outcome measurement. We hypothesize that this combination of technologies will offer superior assessment accuracy compared to traditional methods and provide predictive insights into therapeutical outcomes.

## Introduction

Spasticity is defined as a velocity-dependent increase in muscle stretch resistance [1], which is associated with increased muscle tone. Spasticity is one of the leading causes of impaired motor control and thus significant functional limitations in the upper limb, particularly in cases of Cerebral Palsy (CP), after traumatic brain injury, and following strokes. In the upper limb, the most common patterns include spasticity leading to shoulder adduction, shoulder internal rotation, elbow flexion, forearm pronation, wrist flexion and ulnar deviation, finger flexion, “thumb-in-palm” deformity, clenched fist, and swan-neck deformities of the fingers due to hyperactivity of the intrinsic muscles. If left untreated, spasticity can lead to muscle and later joint contractures, resulting in progressive functional loss of the upper limb and care problems.

The standardized clinical examination of patients with upper limb spasticity includes the assessment of muscle strength grades (MRC scale), as well as the assessment of muscle tone and the extent of spasticity using clinical scales (modified Ashworth Scale (MAS) [2] and Tardieu Scale (TS) [3]), and functional tests including video recordings (Leclercq 2003).

Conservative treatment methods include physiotherapy, occupational therapy, and splint therapy, possibly combined with oral antispastic medication or intrathecal administration of Baclofen®. After exhausting conservative therapy options, and depending on the level of suffering and the patient’s wishes, surgical therapy can be performed. This usually consists of a combination of tendon and nerve procedures. By means of intramuscular lengthening (fractional lengthening), release of the muscle aponeurosis, tendon lengthening (Z-lengthening), or possibly tenotomies, the overactivity of the spastic muscles is reduced, and optimally, the full range of motion of the adjacent joints is restored [4,5]. If this is not successful, additional arthrolysis or even arthrodesis may need to be considered. An essential second pillar of surgical therapy are the so-called hyperselective neurotomies (HSN), which are less commonly studied. In HSN, 2/3 of the nerve fibers innervating a particular muscle are severed to reduce or eliminate the spasticity of that muscle without loss of strength [6–8].

The advances in conservative and especially surgical treatment of spasticity, with the possibility of substantial reduction or elimination of spasticity, necessitate the further development of diagnostic capabilities. To best advise patients regarding optimal therapy, objective, standardized examinations and the collection of short- and long-term therapy results are essential. The scales used in clinical routine to assess spasticity (MAS and TS) are neither precise nor reliable [9] and cannot differentiate between the neural component of spasticity (spasticity) and the non-neural component (contracture).

With the advancement of 3D motion analysis, wireless wearable sensors, and new approaches from robotics and computer technology, various approaches to instrumented spasticity testing have been developed and summarized in a review [9]. To differentiate between spasticity and contracture, both mechanical variables such as joint angles, angular velocities, and joint moments, as well as electrophysiological parameters using surface EMG, should be collected [10,11]. These so-called integrated models often require a very elaborate experimental setup and are therefore not yet used in clinical routine [9]. However, it has been shown that instrumented spasticity testing using surface EMG and motion analysis is superior to MAS and TS [12].

In classical 3D motion analysis, infrared light-reflecting markers are placed on specific anatomical landmarks onto the skin, and their movement in space is captured using infrared cameras. Biomechanical models are then used to calculate joint angles for each instant of a movement. Various models are available for 3D motion analysis of the upper limb. The classical models of the upper limb capture shoulder, elbow, and wrist movements, while specialized hand models [13] capture all joints located distal to the wrist. The so-called U.L.E.M.A. model has already been successfully applied to analyze patients with CP in the upper limb [14].

Another promising approach for precise diagnosis and prediction of surgical outcomes is musculoskeletal modeling and simulation of movements and loads. Based on anatomical information from MRI images, cadaver studies, and 3D motion analysis [15,16], many generic models have already been created. From these generic models, an individual model for each person can be generated by combining body measurements with data from 3D motion analysis and surface EMG (sEMG). Such models mathematically represent the musculoskeletal structure of a specific individual, enabling the calculation of muscle forces, joint loads, muscle tendon lengths, and stretch velocities for specific movements that cannot be directly measured. This approach has already been successfully applied to patients with neurologically induced gait disorders (especially spasticity) [16]. However, there is currently no comparable modeling approach for the upper limb.

The goal of the proposed project is to establish, for the first time, a standardized, objective method for detecting wrist and finger spasticity in the upper limb to improve treatment decisions, conduct adequate outcome analyses, and, using additional musculoskeletal modeling, provide an instrument for predicting surgical outcomes.

## Hypotheses

The combination of 3D motion analysis and sEMG is suitable for objectively detecting spasticity in wrist and finger flexors, providing an effective preoperative assessment tool for surgical therapy strategy and postoperative outcome control.

To test the central hypothesis, the following sub-hypotheses will be investigated

a) Simultaneous recording of sEMG signals from all wrist flexors, extensors, and extrinsic finger extensors and flexors, as well as capturing all joint angles distal to the forearm using 3D motion analysis, is feasible in healthy participants and patients with spasticity.
b) The combination of 3D motion analysis and sEMG measurement allows for a reliable measurement of wrist and finger spasticity.
c) Objective detection of wrist and finger spasticity can be performed within a clinically acceptable timeframe.
d) The proposed methodology is suitable for measuring postoperative improvement of wrist and finger spasticity after hyperselective neurectomy, tendon lengthening, and/ or transposition.
e) A musculoskeletal modeling generated from 3D motion analysis can predict the postoperative outcome after surgical treatment for upper limb spasticity based on preoperative measurements.

## Materials and methods

This study uses a prospective, observational design to evaluate wrist and finger spasticity in healthy participants and patients undergoing surgical treatment for spasticity.

It is a monocentric study (BG Klinik Ludwigshafen, Ludwigshafen, Germany).

Participant recruitment began in February 2023 and is scheduled to conclude in January 2026.

### Ethical approval and trial registration

The study has been approved by the local ethics committee (2020–15528_2).

Clinical trial registration is provided (DRKS00035052).

### Participants

- **Healthy Participants**: 20 arms of healthy participants will be recruited for baseline measurements.
  - **Inclusion criteria:**
    - No previous injuries of the upper extremity
    - No neurological impairments
    - Informed consent
- **Patients**: Patients with upper limb spasticity will be recruited during consultations in a specialized outpatient clinic when presenting for evaluation regarding potential surgical intervention. Clinical assessments in the movement laboratory will be conducted preoperatively (and potentially a second time one week later), 6 months postoperatively, and 1 year postoperatively if surgery is performed. The approximate duration of each measurement is one hour.
  - **Inclusion criteria**:
    - Patients with spasticity of the upper extremity and an indication for surgery
  - **Exclusion criteria:**
    - Severe mental disability (inability to give consent)
    - Botulinum toxin injection in the area of the upper extremity to be treated within the last 6 months

All participants provide written informed consent. If minors are included, written informed consent is obtained from parents or guardians.

Participant data will be stored in a pseudonymized form, ensuring that personal information is collected, shared, and maintained securely to protect confidentiality. Only the principal investigators have access to the key required to trace the pseudonymization, and this access is strictly regulated before, during, and after the trial.

### Equipment

- **Surface EMG**: sEMG electrodes will be placed to capture muscle activity from wrist flexors (Flexor carpi ulnaris (FCU), Flexor carpi radialis (FCR)), wrist extensors (Extensor carpi radialis longus (ECRL), Extensor carpi radialis brevis (ECRB), Extensor carpi ulnaris (ECU)), extrinsic finger flexors (Flexor digitorum superficialis (FDS) 2–5, Flexor digitorum profundus (FDP) 2–5, extrinsic finger extensors (Extensor digitorum communis (EDC)), and extrinsic thumb flexor and extensor (Flexor pollicis longus (FPL), Extensor pollicis longus (EPL)). EMG signals will be normalized to maximum voluntary isometric contraction (MVIC) recorded at the beginning of the measurement session for each respective muscle group.
- **3D Motion Analysis**: Infrared light-reflecting markers will be placed on anatomical landmarks to capture joint angles using 3D motion analysis. Two hand marker sets [17–19] will be employed for detailed analysis of wrist and finger movements.

Target parameters are flexion/extension in the metacarpophalangeal joints (MCP) 1-5, flexion/extension in the interphalangeal joint (IP), flexion/extension in the proximal interphalangeal joints (PIP) 2-5, and distal interphalangeal joints (DIP) 2-5. The proposed hand models captures MCP 2-5 with 2 degrees of freedom (extension/flexion, abduction/adduction), IP, PIP, and DIP each with one degree of freedom (extension/flexion), and the thumb saddle joint (MCP 1) with 3 degrees of freedom (extension/flexion, abduction/adduction, rotation).

Using the U.L.E.M.A. model [14], wrist flexion and extension will be captured, modeling the wrist with three degrees of freedom (wrist flexion/extension, wrist radial/ulnar deviation, forearm pronation/supination).

The angular velocity is calculated as the first derivative of the joint angles recorded at each time point, and acceleration as the second derivative.

### Detailed work plan

The instrumented assessment of wrist and finger spasticity will be analogous to clinical spasticity testing, capturing MAS and TS through slow and fast passive stretching of the muscle groups to be tested. During this process, sEMG signals and relevant joint angles will be measured using 3D motion analysis. Concurrently, subjective MAS and TS values will be recorded.

**Fig 1:**
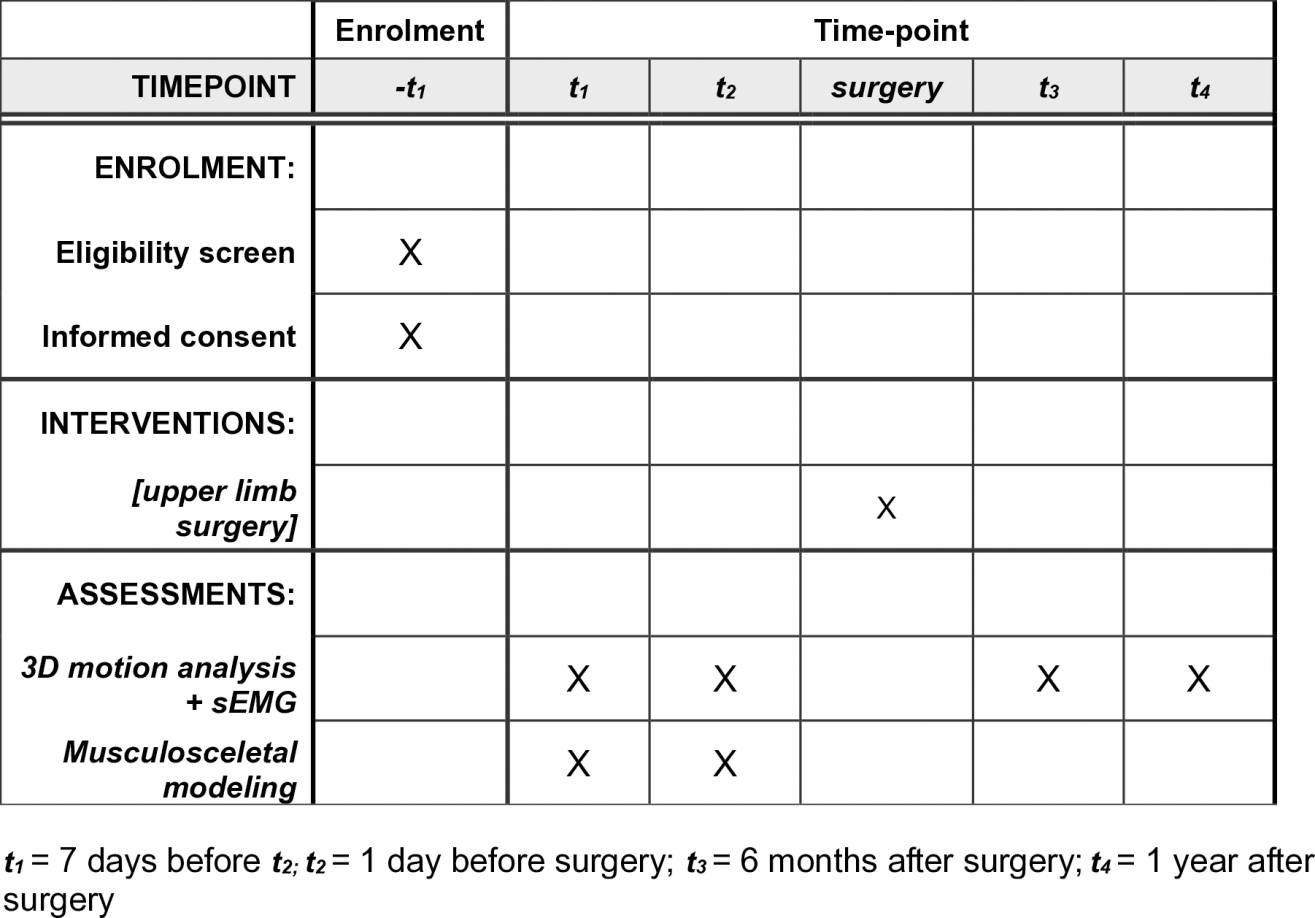
SPIRIT schedule: t1 = 7 days before t2; t2  = 1 day before surgery; t3 = 6 months after surgery; t4 = 1 year after surgery.

1. **Surface EMG and Motion Analysis**: sEMG signals and joint angles will be recorded during slow (30°/s) and fast (180°/s) passive stretching of the muscle groups [20]. A 5-second pause will be maintained between each repetition. The protocol includes the following passive stretches:
  Wrist extension with a clenched fist (particularly targeting FCU, FCR)
  Wrist extension with open fingers (targeting FDS, FDP, FCU, FCR)
  Thumb extension with the wrist in a neutral position (targeting FPL)
2. **Measurement Procedure**: Stretching will be performed manually to ensure clinical applicability. Measurements will be taken with a metronome to maintain speed consistency and include inter-rater reliability testing.
3. **Musculoskeletal Modeling**: A musculoskeletal model generated from 3D motion analysis will be applied to predict postoperative outcomes based on preoperative measurements. Specifically, this involves using preoperative experimental data from the patient and an individually scaled musculoskeletal model, into which the planned surgical procedure (e.g., muscle-tendon lengthening) is incorporated. This allows for the simulation of potential surgical outcomes, such as changes in joint range of motion or the patient’s ability to generate functional movement.
  For musculoskeletal modeling, the dynamic upper extremity model developed by Saul and Holzbaur [21] was selected due to its high level of anatomical detail, ranging from the thorax to the wrist. The model includes 22 segments and 15 degrees of freedom. Additionally, it represents 32 muscles using 50 Hill-type muscle-tendon actuators. Forward dynamics simulations will be conducted using EMG data as input.
4. **Measurement Time Points** (Fig 1):
  **HEALTHY PARTICIPANTS:** Two measurements, 7 days apart (***t***_***1***_***, t***_***2***_**)**
  **PATIENT:** Four measurement points, if possible: 8 days preoperative (t_1_), 1 day preoperative (t_2_), 6 months postoperative (t_3_), and 1 year postoperative (t_4_).

5 **Outcome Parameters:** The following EMG parameters will be recorded for all specified muscles:
  ▫ **MVIC:** The highest EMG activity within a defined period of 250ms during maximum voluntary isometric muscle contraction.
  ▫ **EMG LV:** Average EMG activity during slow (30°/s) passive stretch within a defined period (starting 200ms prior reaching maximum stretching velocity until 90% of the maximal range of motion is achieved [12]).
  ▫ **EMG HV**: Average EMG activity during fast (180°/s) passive stretch within a defined period (starting 200ms prior reaching maximum stretching velocity until 90% of the maximal range of motion is achieved [12]).
  **EMGchange:** EMG HV - EMG LV [12].
6 **Covariates:**
  ▫ **Angular Velocity** (calculated from 3D motion analysis)
  ▫ **Acceleration** (calculated from 3D motion analysis)

### Sample size calculation

Based on our preliminary work, the following effect size and resulting sample size were calculated for the primary outcome parameter, EMGchange, during passive stretch of the biceps brachii in patients with elbow flexor spasticity (one-sided, α = 0.05, power (1–β) = 0.8) compared to healthy participants. (Table 1)

**Table 1:** Sample size calculation based on the primary outcome parameter (EMGchange (%MVIC)); SD = standard deviation.

Parameter	Mean (patient)	SD (patient)	Mean (healthy)	SD (healthy)	Effect Size	Sample Size per group
EMGchange	19.1	21.1	0.04	0.1	0.91	10

The sample size calculation for the pre-/post-surgical comparison is based on our preliminary data. In a group of 7 patients with elbow flexor spasticity who underwent hyperselective neurectomy (HSN) of the elbow flexors as a surgical treatment, we were able to perform objective spasticity testing of the elbow. For the primary outcome parameter, EMGchange, the following effect size and resulting sample size were determined (one-sided, α = 0.05, power (1-β) = 0.8) (Table 2):

**Table 2:** Sample size calculation for a pre-/post-analysis based on the primary outcome parameter (EMGchange (%MVIC)) in 7 patients with elbow flexor spasticity who underwent hyperselective neurectomy (HSN) of the elbow flexors; SD = standard deviation.

Parameter	Mean preop	SD preop	Mean postop	SD postop	Effect Size	Sample Size
EMGchange (n = 7)	21.7	23.7	7.4	4.2	0.65	16

### Statistical analysis

To determine the reliability of the proposed instrumented assessment protocol, a total of 20 patients with spasticity will be included. These patients must not have undergone prior surgery distal to the elbow and must not have received botulinum toxin injections within the past 6 months. A preoperative measurement will be conducted twice for each patient within 1 week to assess the repeatability (test-retest reliability) of the proposed assessment. By correlating the outcome parameters of the two measurement time points, the Intraclass Correlation Coefficient (ICC, interval-scaled data) will be calculated. Similarly, the ICC for healthy participants will be calculated based on the two measurements 1 week apart. The inter-rater reliability will be determined for healthy participants and for patients with spasticity using the measurement at the first time point each. For this purpose, all spasticity assessments will be conducted by two trained examiners. The ICC will then be calculated by correlating the outcome parameters. Standard Error of Measurement (SEM) and Minimal Detectable Difference (MDD) will be reported.

The 20 arms of the healthy participants may include both arms from the same individual. However, measurements will be performed on two separate days. We assume that each arm functions independently without influencing the other side and therefore treat all arms as independent samples, regardless of whether data from both arms of a single participant are included.

A validity analysis in the strict sense is not possible for the proposed methodology, as there is currently no non-invasive valid measurement technique for assessing spasticity. Alternatively, in clinical practice, the subjective scales MAS and TS are used with moderate reliability. Accordingly, the correlation of the determined spasticity parameters by the instrumented assessment method with both scales will be calculated. For this, the Spearman rank correlation coefficient (minimum ordinal data) will be used.

The study design concerning pre-/post treatment analysis corresponds to an experimental prospective cohort study. Initially, the normal distribution of the interval-scaled parameters will be confirmed (Kolmogorov-Smirnov test and visual inspection using histograms).

After analyzing the preoperative and 6 months postoperative measurements from a minimum of 15 patients, a first interim analysis will be conducted using a paired two-sample t-test to determine any significant changes in the defined outcome parameters (EMG LV, EMG HV, EMGchange, extension deficit for wrist flexors (FCR, FCU), and finger flexors (FPL, FDP, and FDS)). If normal distribution is not confirmed, the Wilcoxon test will be applied.

Upon completion of all postoperative measurements, changes in the defined outcome parameters (EMG LV, EMG HV, EMGchange, extension deficit for wrist flexors (FCR, FCU), and finger flexors (FPL, FDP, and FDS)) across the three measurements (preoperative, 6 months postoperative, 1 year postoperative) will be examined using one-way repeated measures ANOVA. If histogram analysis and the Kolmogorov-Smirnov test indicate that the data are not normally distributed, the Friedman test will be used as a non-parametric alternative to ANOVA for comparing the three measurements.

The significance level α is set at 0.05. For significant differences, a post hoc paired t-test will be conducted, with α adjusted for multiple testing using the Bonferroni-Holm correction.

### Significance of the project

The development of an objective method for assessing wrist and finger spasticity will enhance therapeutic decision-making and postoperative evaluation. This approach aims to minimize the need for multiple surgeries and prolonged hospital stays by providing a comprehensive diagnostic and outcome measurement tool. The long-term goal is to establish a multicentric database for continuous evaluation of surgical interventions.

## Discussion

The project aims to develop an instrumented assessment method to differentiate between wrist and finger spasticity. As a secondary goal, a simulation of potential surgical outcomes is planned. If the developed methodology is successfully implemented, patients with upper limb spasticity could be optimally treated with a single surgical intervention that corrects all deformities, within the framework of so-called “single-event-multilevel surgeries.” This approach could spare patients from previous clinical testing through botulinum toxin injections and multiple surgeries with extended hospital stays by providing a clear diagnosis and treatment plan. Additionally, the instrumented outcome measure would serve as a crucial quality control for the surgical therapy.

The long-term goal is to establish a large multicentric database. This will allow for the continued investigation and assessment of individual surgical steps, despite the varying combinations of surgical procedures, and enable an evaluation of their effectiveness over time. It will offer patients a significant improvement in independence and, consequently, quality of life.

### Study limitations

There are some study limitations that have to be addressed and taken into account when interpreting the data. First there is no consensus on how to normalize EMG data in patients with spasticity. MVIC normalization is most commonly performed, although its well-known limitations in patients with reduced voluntary motor control. There exist no SENIAM guidelines for sEMG electrode placement on the forearm. The muscle bellies are very close together and partially overlap, suggesting significant cross-talk. As a result, it is unclear how reliably the sEMG data can be interpreted on the forearm. All electrode and marker placements will be documented with photographs. The recorded sEMG data will be visually inspected and assessed for potential cross-talk. The measurement effort for each individual patient is approximately 1 hour under the study protocol and needs to be minimized for implementation in clinical routine. Due to the low number of eligible patients, the study group will be heterogeneous and include individuals with CP post-stroke, and post-hemorrhagic spasticity. This has to be considered when interpreting data. Concerning the spasticity itself, there are no differences expected between patient groups. When evaluating upper limb function differences in learned and lost (post-stroke, post-hemorrhage) versus never learned (CP) movement patterns to due disturbed volitional motor control must be considered. At least for test-retest reliability in patients the additional effort for a second preoperative measurement might be too high and there might be not enough patients to be included for this special testing. On the other hand, spasticity is highly influenced by daily conditions, environment, emotional state, and temperature – raising the question of whether a test-retest measurement truly assesses the method itself or merely reflects fluctuations in the patient’s condition.

## Conclusion

This study will provide new insights into the efficacy of instrumented assessment tools for spasticity and their potential impact on surgical treatment strategies. The integration of 3D motion analysis, surface EMG, and musculoskeletal modeling represents a significant advancement in spasticity assessment, with the potential to improve patient outcomes and optimize treatment decisions.

## Supporting information

S1 File SPIRIT 2013 Checklist: Recommended items to address in a clinical trial protocol and related documents*. (DOC)

S2 File Studienprotokoll_V4_Amendment. (DOCX)

S3 File Studienprotokoll_V4_Amendment_engl. (DOCX)

S4 File Supplement_patientdata_samplesizecalculation. (XLSX)
