# Supplementary material for: An instrumented approach to quantify wrist and finger flexor spasticity: A study protocol
Source: PLoS One. 2025 Jul 31;20(7):e0328528. doi: 10.1371/journal.pone.0328528 (PMC12312980; doi:10.1371/journal.pone.0328528)
Supplement: S2 File — (DOCX) [file pone.0328528.s002.docx]

**Studienprotokoll**

**Projekttitel:**

Prospektive objektive Erfassung der postoperativen Ergebnisse an der oberen Extremität bei Patienten mit Spastik hinsichtlich Schmerzen, Spastik und Bewegungsausmaß

**Studienleiter:**

PD Dr. med. Mirjam Thielen und
Ursula Trinler, PhD
BG Klinik Ludwigshafen
(BG Kliniken Ludwigshafen und Tübingen gGmbH)
Ludwig-Guttmann-Str. 13
67071 Ludwigshafen

**Weitere an der Studie beteiligte Wissenschaftler/innen:**

apl. Prof. Dr. med. Leila Harhaus

Anna Pennekamp

Julia Glaser

**Beteiligte Einrichtungen:**

BG Klinik Ludwigshafen
Ludwig-Guttmann-Str. 13
67071 Ludwigshafen

**Registrierung in einem öffentlich zugänglichen Studienregister:**

---

**Datum / Version:**

11.08.2022 / Version 4

**Zusammenfassung des Projekts**

Die Spastik, ein geschwindigkeitsabhängig gesteigerter Dehnungswiderstand der Muskulatur, stellt eine der führenden Ursachen für eine gestörte Bewegungskontrolle im Bereich der oberen Extremitäten, insbesondere bei Patienten mit infantiler Cerebralparese, Patienten nach Hirnblutungen, Patienten nach Schlaganfall und bei spastischer inkompletter Tetraparese aufgrund eines hohen Querschnittsyndroms, dar. Unbehandelt führt die Spastik zu Muskel- und später Gelenkkontrakturen.

Nach Ausschöpfung der konservativen Therapiemaßnahmen, sowie bei entsprechendem Leidensdruck und Wunsch der Patienten kann eine operative Therapie durchgeführt werden, die sich in der Regel aus einer Kombination von Muskel- / Sehnenverlängerungen sowie Nerveneingriffen zusammensetzt. Bei den Nerveneingriffen handelt es sich um sogenannte hyperselektive Neurotomien (HSN), bei denen 2/3, der einen bestimmten Muskel innervierenden Nervenfasern, selektiv durchtrennt werden, um ohne Kraftverlust die Spastik dieses Muskels zu eliminieren.

Ziel dieser Studie ist es nun erstmalig prospektiv alle Patienten mit einem spastischen Krankheitsbild an der oberen Extremität, die aufgrund dieser Diagnose operativ in der BG Unfallklinik Ludwigshafen behandelt werden, in einer Datenbank zu erfassen und durch die Dokumentation und Auswertung der objektiven Untersuchungsergebnisse erstmalig prospektive Kurz-, Mittel und Langzeitergebnisse der verschiedenen operativen Therapieverfahren zu erhalten.

1. **Wissenschaftlicher Hintergrund**

Spastik ist definiert als ein geschwindigkeitsabhängig gesteigerter Dehnungswiderstand der Muskulatur [1], der mit einem erhöhten Muskeltonus einhergeht [2]. Die Spastik stellt eine der führenden Ursachen für eine gestörte Bewegungskontrolle und damit deutliche Funktionseinschränkung im Bereich der oberen Extremitäten, insbesondere bei Patienten mit bilateraler und unilateraler spastischer infantiler Cerebralparese, Patienten nach Hirnblutungen, Patienten nach Schlaganfall und bei Patienten mit spastischer Tetraparese bei hohem Querschnittsyndrom, dar [3-5]. Im Bereich der oberen Extremität sind die häufigsten Muster eine Schulteradduktion, Schulterinnenrotation, Ellenbogenflexion, Unterarmpronation, Handgelenkflexion und -ulnardeviation, „Thumb-in-palm“ Deformität, clenched fist, sowie Schwanenhalsdeformitäten der Finger bei Intrinsic+ Syndrom. Unbehandelt führt die Spastik zu Muskel- und später Gelenkkontrakturen und damit zu einem progredienten Funktionsverlust der oberen Extremität sowie Pflegeproblemen. [3, 6, 7]

Die standardisierte Untersuchung dieser Patienten umfasst eine Kraftmessung, die Messung des Muskeltonus und des Ausmaßes der Spastik (modifizierte Ashworth Skala [8] und modifizierte Tardieu Skala [9, 10]) sowie funktionelle Tests inklusive Videoaufnahmen [6, 11, 12]. Die Methode der 3D Bewegungsanalyse erlaubt uns inzwischen auch für die oberen Extremitäten zusätzlich objektive Untersuchungen der Bewegungsausmaße bei Alltagsbewegungen durchzuführen [13-16]. Die instrumentierte Spastik-Prüfung mittels Oberflächen-EMG und Bewegungsanalyse ist der Ashworth Skala [8] sowie der Tardieu Skala [9, 10] überlegen [17-20].

Als konservative Therapiemethoden stehen Physiotherapie, Ergotherapie und Schienenbehandlung, ggf. kombiniert mit tonushemmender oraler Medikation oder intrathekaler Verabreichung von Baclofen® zur Verfügung. Nach Ausschöpfung der konservativen Therapiemaßnahmen, sowie bei entsprechendem Leidensdruck und Wunsch der Patienten kann eine operative Therapie durchgeführt werden. Die operative Therapie setzt sich in der Regel aus einer Kombination von Sehnen- und Nerveneingriffen zusammen. Mittels intramuskulärer Verlängerung (fraktionierte Verlängerung), Release der Muskelaponeurose, Sehnenverlängerungen oder ggf. auch Tenotomien wird die Überaktivität der spastischen Muskulatur reduziert und optimaler Weise der vollständige Bewegungsumfang der angrenzenden Gelenke wiederhergestellt [21-40]. Sollte dies nicht gelingen, muss ggf. eine zusätzliche Arthrolyse oder sogar Arthrodese erwogen werden [41, 42]. Als zweiter Pfeiler der operativen Therapie gelten die sog. hyperselektiven Neurotomien (HSN), bei denen 2/3, der einen bestimmten Muskel innervierenden, Nervenfasern durchtrennt werden, um ohne Kraftverlust die Spastik dieses Muskels zu eliminieren [3, 43-48].

Obwohl unter den ausgewiesenen Experten auf dem Gebiet der spastischen Handchirurgie Einigkeit über die o.g. Therapieverfahren sowie Diagnostik herrscht, gibt es unseres Wissens bisher keine Studie, die die Behandlungserfolge nach operativer Therapie der oberen Extremität bei verschiedenen spastischen Krankheitsbildern prospektiv objektiv (mittels 3D Bewegungsanalyse) kurz-, mittel- und langfristig erfasst.

Literatur:

1. Lance, J.W., *What is spasticity?* Lancet, 1990. **335**(8689): p. 606.

2. Sanger, T.D., et al., *Classification and definition of disorders causing hypertonia in childhood.* Pediatrics, 2003. **111**(1): p. e89-97.

3. Mikalef, P. and D. Power, *The role of neurectomy in the management of spasticity of the upper limb.* EFORT Open Rev, 2017. **2**(11): p. 469-473.

4. Tranchida, G.V. and A. Van Heest, *Preferred options and evidence for upper limb surgery for spasticity in cerebral palsy, stroke, and brain injury.* J Hand Surg Eur Vol, 2020. **45**(1): p. 34-42.

5. Angulo-Parker, F.J. and J.M. Adkinson, *Common Etiologies of Upper Extremity Spasticity.* Hand Clin, 2018. **34**(4): p. 437-443.

6. Leclercq, C., *General assessment of the upper limb.* Hand Clin, 2003. **19**(4): p. 557-64.

7. Rhee, P.C., *Surgical Management of the Spastic Forearm, Wrist, and Hand: Evidence-Based Treatment Recommendations: A Critical Analysis Review.* JBJS Rev, 2019. **7**(7): p. e5.

8. Bohannon, R.W. and M.B. Smith, *Interrater reliability of a modified Ashworth scale of muscle spasticity.* Phys Ther, 1987. **67**(2): p. 206-7.

9. Mackey, A.H., et al., *Intraobserver reliability of the modified Tardieu scale in the upper limb of children with hemiplegia.* Dev Med Child Neurol, 2004. **46**(4): p. 267-72.

10. Mehrholz, J., et al., *Reliability of the Modified Tardieu Scale and the Modified Ashworth Scale in adult patients with severe brain injury: a comparison study.* Clin Rehabil, 2005. **19**(7): p. 751-9.

11. Waters, P.M., et al., *Interobserver and intraobserver reliability of therapist-assisted videotaped evaluations of upper-limb hemiplegia.* J Hand Surg Am, 2004. **29**(2): p. 328-34.

12. Carlson, M.G., et al., *Impact of video review on surgical procedure determination for patients with cerebral palsy.* J Hand Surg Am, 2009. **34**(7): p. 1225-31.

13. Jaspers, E., et al., *Review of quantitative measurements of upper limb movements in hemiplegic cerebral palsy.* Gait Posture, 2009. **30**(4): p. 395-404.

14. Jaspers, E., et al., *The reliability of upper limb kinematics in children with hemiplegic cerebral palsy.* Gait Posture, 2011. **33**(4): p. 568-75.

15. Jaspers, E., et al., *Upper limb kinematics: development and reliability of a clinical protocol for children.* Gait Posture, 2011. **33**(2): p. 279-85.

16. Bar-On, L., et al., *A clinical measurement to quantify spasticity in children with cerebral palsy by integration of multidimensional signals.* Gait Posture, 2013. **38**(1): p. 141-7.

17. Bar-On, L., et al., *Manually controlled instrumented spasticity assessments: a systematic review of psychometric properties.* Dev Med Child Neurol, 2014. **56**(10): p. 932-50.

18. Bar-On, L., et al., *Is an instrumented spasticity assessment an improvement over clinical spasticity scales in assessing and predicting the response to integrated botulinum toxin type a treatment in children with cerebral palsy?* Arch Phys Med Rehabil, 2014. **95**(3): p. 515-23.

19. McGibbon, C.A., et al., *Elbow spasticity during passive stretch-reflex: clinical evaluation using a wearable sensor system.* J Neuroeng Rehabil, 2013. **10**(1): p. 61.

20. Keenan, M.A., T.T. Haider, and L.R. Stone, *Dynamic electromyography to assess elbow spasticity.* J Hand Surg Am, 1990. **15**(4): p. 607-14.

21. Tafti, M.A., S.C. Cramer, and R. Gupta, *Orthopaedic management of the upper extremity of stroke patients.* J Am Acad Orthop Surg, 2008. **16**(8): p. 462-70.

22. Namdari, S., et al., *Shoulder tenotomies to improve passive motion and relieve pain in patients with spastic hemiplegia after upper motor neuron injury.* J Shoulder Elbow Surg, 2011. **20**(5): p. 802-6.

23. Namdari, S., et al., *Outcomes of tendon fractional lengthenings to improve shoulder function in patients with spastic hemiparesis.* J Shoulder Elbow Surg, 2012. **21**(5): p. 691-8.

24. Carlson, M.G., et al., *Early results of surgical intervention for elbow deformity in cerebral palsy based on degree of contracture.* J Hand Surg Am, 2012. **37**(8): p. 1665-71.

25. Dy, C.J., et al., *Long-term results following surgical treatment of elbow deformity in patients with cerebral palsy.* J Hand Surg Am, 2013. **38**(12): p. 2432-6.

26. Anakwenze, O.A., et al., *Myotendinous lengthening of the elbow flexor muscles to improve active motion in patients with elbow spasticity following brain injury.* J Shoulder Elbow Surg, 2013. **22**(3): p. 318-22.

27. Keenan, M.A., et al., *Results of fractional lengthening of the finger flexors in adults with upper extremity spasticity.* J Hand Surg Am, 1987. **12**(4): p. 575-81.

28. Van Heest, A.E., et al., *The supination effect of tendon transfer of the flexor carpi ulnaris to the extensor carpi radialis brevis or longus: a cadaveric study.* J Hand Surg Am, 1999. **24**(5): p. 1091-6.

29. Van Heest, A.E., J.H. House, and C. Cariello, *Upper extremity surgical treatment of cerebral palsy.* J Hand Surg Am, 1999. **24**(2): p. 323-30.

30. Van Heest, A.E., et al., *Tendon transfer surgery in upper-extremity cerebral palsy is more effective than botulinum toxin injections or regular, ongoing therapy.* J Bone Joint Surg Am, 2015. **97**(7): p. 529-36.

31. Van Heest, A., et al., *Follow-up motion laboratory analysis for patients with spastic hemiplegia due to cerebral palsy: analysis of the flexor carpi ulnaris firing pattern before and after tendon transfer surgery.* J Hand Surg Am, 2010. **35**(2): p. 284-90.

32. Thevenin-Lemoine, C., et al., *Flexor origin slide for contracture of spastic finger flexor muscles: a retrospective study.* J Bone Joint Surg Am, 2013. **95**(5): p. 446-53.

33. Inglis, A.E. and W. Cooper, *Release of the flexor-pronator origin for flexion deformities of the hand and wrist in spastic paralysis. A study of eighteen cases.* J Bone Joint Surg Am, 1966. **48**(5): p. 847-57.

34. Braun, R.M., V. Mooney, and V.L. Nickel, *Flexor-origin release for pronation-flexion deformity of the forearm and hand in the stroke patient. An evaluation of the early results in eighteen patients.* J Bone Joint Surg Am, 1970. **52**(5): p. 907-20.

35. El-Said, N.S., *Selective release of the flexor origin with transfer of flexor carpi ulnaris in cerebral palsy.* J Bone Joint Surg Br, 2001. **83**(2): p. 259-62.

36. Peraut, E., et al., *Results and complications of superficialis-to-profundus tendon transfer in brain-damaged patients, a series of 26 patients.* Orthop Traumatol Surg Res, 2018. **104**(1): p. 121-126.

37. Pomerance, J.F. and M.A. Keenan, *Correction of severe spastic flexion contractures in the nonfunctional hand.* J Hand Surg Am, 1996. **21**(5): p. 828-33.

38. Davids, J.R., et al., *Validation of the Shriners Hospital for Children Upper Extremity Evaluation (SHUEE) for children with hemiplegic cerebral palsy.* J Bone Joint Surg Am, 2006. **88**(2): p. 326-33.

39. Davids, J.R., et al., *Surgical management of thumb deformity in children with hemiplegic-type cerebral palsy.* J Pediatr Orthop, 2009. **29**(5): p. 504-10.

40. Alewijnse, J.V., M.J. Smeulders, and M. Kreulen, *Short-term and Long-term Clinical Results of the Surgical Correction of Thumb-in-Palm Deformity in Patients With Cerebral Palsy.* J Pediatr Orthop, 2015. **35**(8): p. 825-30.

41. Van Heest, A.E. and D. Strothman, *Wrist arthrodesis in cerebral palsy.* J Hand Surg Am, 2009. **34**(7): p. 1216-24.

42. Rayan, G.M. and B.T. Young, *Arthrodesis of the spastic wrist.* J Hand Surg Am, 1999. **24**(5): p. 944-52.

43. Gras, M. and C. Leclercq, *Spasticity and hyperselective neurectomy in the upper limb.* Hand Surg Rehabil, 2017. **36**(6): p. 391-401.

44. Kwak, K.W., et al., *Surgical results of selective median neurotomy for wrist and finger spasticity.* J Korean Neurosurg Soc, 2011. **50**(2): p. 95-8.

45. Puligopu, A.K. and A.K. Purohit, *Outcome of selective motor fasciculotomy in the treatment of upper limb spasticity.* J Pediatr Neurosci, 2011. **6**(Suppl 1): p. S118-25.

46. Maarrawi, J., et al., *Long-term functional results of selective peripheral neurotomy for the treatment of spastic upper limb: prospective study in 31 patients.* J Neurosurg, 2006. **104**(2): p. 215-25.

47. Purohit, A.K., et al., *Selective musculocutaneous fasciculotomy for spastic elbow in cerebral palsy: a preliminary study.* Acta Neurochir (Wien), 1998. **140**(5): p. 473-8.

48. Brunelli, G. and F. Brunelli, *Partial selective denervation in spastic palsies (hyponeurotization).* Microsurgery, 1983. **4**(4): p. 221-4.

1. Projektziele

Ziel dieser Studie ist es nun erstmalig prospektiv alle Patienten mit einem spastischen Krankheitsbild an der oberen Extremität, die aufgrund dieser Diagnose operativ in der BG Unfallklinik Ludwigshafen operiert werden, in einer Datenbank zu erfassen und durch die Dokumentation und Auswertung der objektiven Untersuchungsergebnisse, Kurz-, Mittel und Langzeitergebnisse der verschiedenen operativen Therapieverfahren zu untersuchen.

1. Zielgrößen

- primäre Zielgrößen:

jeweils zu allen Messzeitpunkten (1 Woche präoperativ, 6 Monate postoperativ, 1 Jahr postoperativ, 3 Jahre postoperativ, 5 Jahre postoperativ, 10 Jahre postoperativ):

- Schmerz auf der visuellen Analogskala
- Schmerzfragebogen „Pain-Detect“
- Ausmaß der Spastik der betroffenen Muskelgruppen auf der modifizierten Ashworth Skala, auf der Tardieu-Skala sowie mittels instrumentierter Spastik-Prüfung (Oberflächen-EMG + Bewegungsanalyse)
- Passive Bewegungsausmaße der betroffenen Gelenke (Ellenbogen, Handgelenk, Metacarpophalangealgelenke und Interphalangealgelenke) der oberen Extremität
- Aktive Bewegungsausmaße der betroffenen Gelenke der oberen Extremität
- Muskelkraft (nach Janda) der betroffenen Muskelgruppen der oberen Extremität
- Aktive Bewegungsausmaße der betroffenen Gelenke (Ellenbogenextension / -flexion, Handgelenkextension /-flexion, Handgelenkradial- /-ulnarduktion, Unterarmpro- /-supination, Metacarpophalangealgelenkextension /-flexion und Interphalangealgelenkextension /-flexion) der oberen Extremität im Rahmen der 3D-Bewegungsanalyse bei gerichteten Gelenkbewegungen sowie Alltagsbewegungen (Hand zu Mund, Hand zu Kopf, Hand zu kontralateraler Schulter) und Greifbewegungen (auf Schulterhöhe vorwärts, nach oben (Augenhöhe), auf Schulterhöhe seitwärts, Ball greifen, horizontalen Zylinder greifen, vertikalen Zylinder greifen)
- Bei Patienten mit der Diagnose infantile Cerebralparese: MACS-Score (Manual Ability Classification System)

1. Studienpopulation

- Einschlusskriterien:
  - Patienten mit Spastik an der oberen Extremität und Operationsindikation zur Behandlung dieses Krankheitsbildes
- Ausschlusskriterien:
  - schwere geistige Behinderung (Unfähigkeit zur Einwilligung (Betreuung), Unfähigkeit zur kognitiven Umsetzung von vorgegebenen gezielten Bewegungsaufgaben) , Non-Compliance und / oder Unfähigkeit der Einwilligung
  - Botulinumtoxininjektion im Bereich der zu behandelnden oberen Extremität innerhalb der letzten 6 Monate
  - nicht-einwilligungsfähige Patienten
- Anzahl der Studienteilnehmer:

Nach Möglichkeit sollen ab sofort alle Pat. mit spastischem Krankheitsbild an der oberen Extremität, die aufgrund dieser Diagnose in der BG Unfallklinik Ludwigshafen eine operative Therapie erhalten, prospektiv in die Datenbank aufgenommen werden.

- Rekrutierungsmaßnahmen:
  Die Patienten werden im Rahmen der ambulanten Vorstellung in der Spezialsprechstunde, bei der die Operationsindikation gestellt wird, rekrutiert.

Kontrollgruppe (Probanden):

10 einwilligungsfähige Erwachsene ohne Vorerkrankungen im Bereich der oberen Extremitäten

1. Methodik und Durchführung

Es handelt sich um eine monozentrische Studie. Die Aufklärung und Einholung der Einwilligung erfolgt im Rahmen der ambulanten Vorstellung der Patienten in der Spezialsprechstunde, bei der die Operationsindikation gestellt wird. Die Daten werden alle prospektiv erhoben.

Im Rahmen der Therapieplanung und postoperativen Kontrolle werden bei den Patienten mit Spastik standardisierte instrumentelle Bewegungsanalysen und klinische Untersuchungen inkl. Erhebung des Schmerzniveaus in regelmäßigen Abständen (1 Woche präoperativ, 6 Monate postoperativ, 1 Jahr postoperativ, 3 Jahre postoperativ, 5 Jahre postoperativ, 10 Jahre postoperativ) durchgeführt. Dies erfolgt im Rahmen der klinischen Routine. Alle bei der Auswertung der Studie benötigten Daten werden im Rahmen dieser klinischen Routineuntersuchungen erhoben. Aus diesem Grund kommen im Rahmen dieser Studie keine zusätzlichen Untersuchungen auf die Patienten zu. Es erfolgt lediglich die Pseudonymisierung und Auswertung der routinemäßig zu erhebenden Daten in einer Datenbank.

- - Auflistung/Beschreibung der zu erfassenden Daten

jeweils zu allen Messzeitpunkten (1 Woche präoperativ, 6 Monate postoperativ,1 Jahr postoperativ, 3 Jahre postoperativ, 5 Jahre postoperativ, 10 Jahre postoperativ):

- Schmerz auf der visuellen Analogskala
- Schmerzfragebogen „Pain detect“
- Ausmaß der Spastik der betroffenen Muskelgruppen auf der modifizierten Ashworth Skala, auf der Tardieu-Skala sowie mittels instrumentierter Spastik-Prüfung (Oberflächen-EMG + Bewegungsanalyse)
- Passive Bewegungsausmaße der betroffenen Gelenke der oberen Extremität
- Aktive Bewegungsausmaße der betroffenen Gelenke der oberen Extremität
- Muskelkraft (nach Janda) der betroffenen Muskelgruppen der oberen Extremität
- Aktive Bewegungsausmaße der betroffenen Gelenke der oberen Extremität im Rahmen der 3D-Bewegungsanalyse bei gerichteten Gelenkbewegungen sowie Alltagsbewegungen (Hand zu Mund, Hand zu Kopf, Hand zu kontralateraler Schulter) und Greifbewegungen (auf Schulterhöhe vorwärts, nach oben (Augenhöhe), auf Schulterhöhe seitwärts, Ball greifen, horizontaler Zylinder greifen, vertikaler Zylinder greifen)
- Bei Patienten mit der Diagnose infantile Cerebralparese: MACS-Score (Manual Ability Classification System)

Markerset und EMG Elektroden


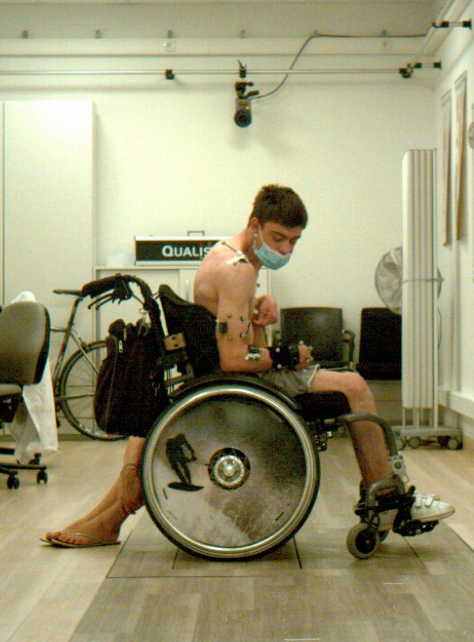

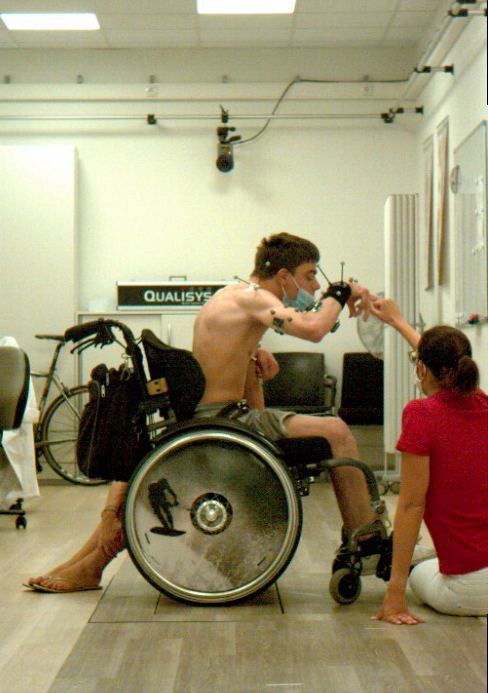

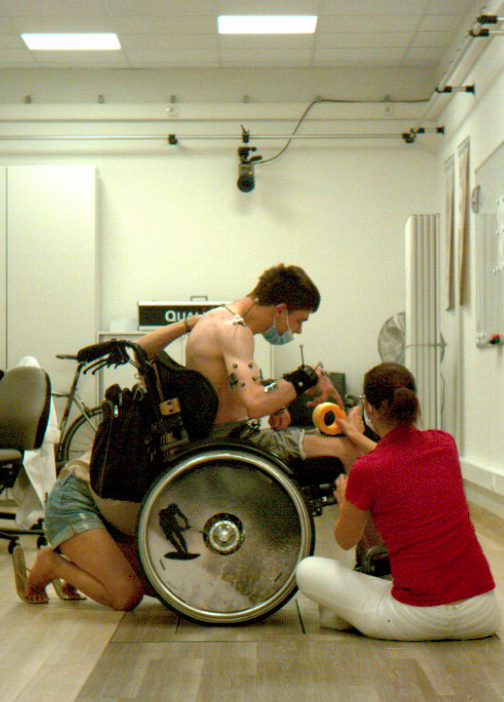


Greifbewegung nach oben auf Augenhöhe

Greifbewegung nach horizontalem Zylinder


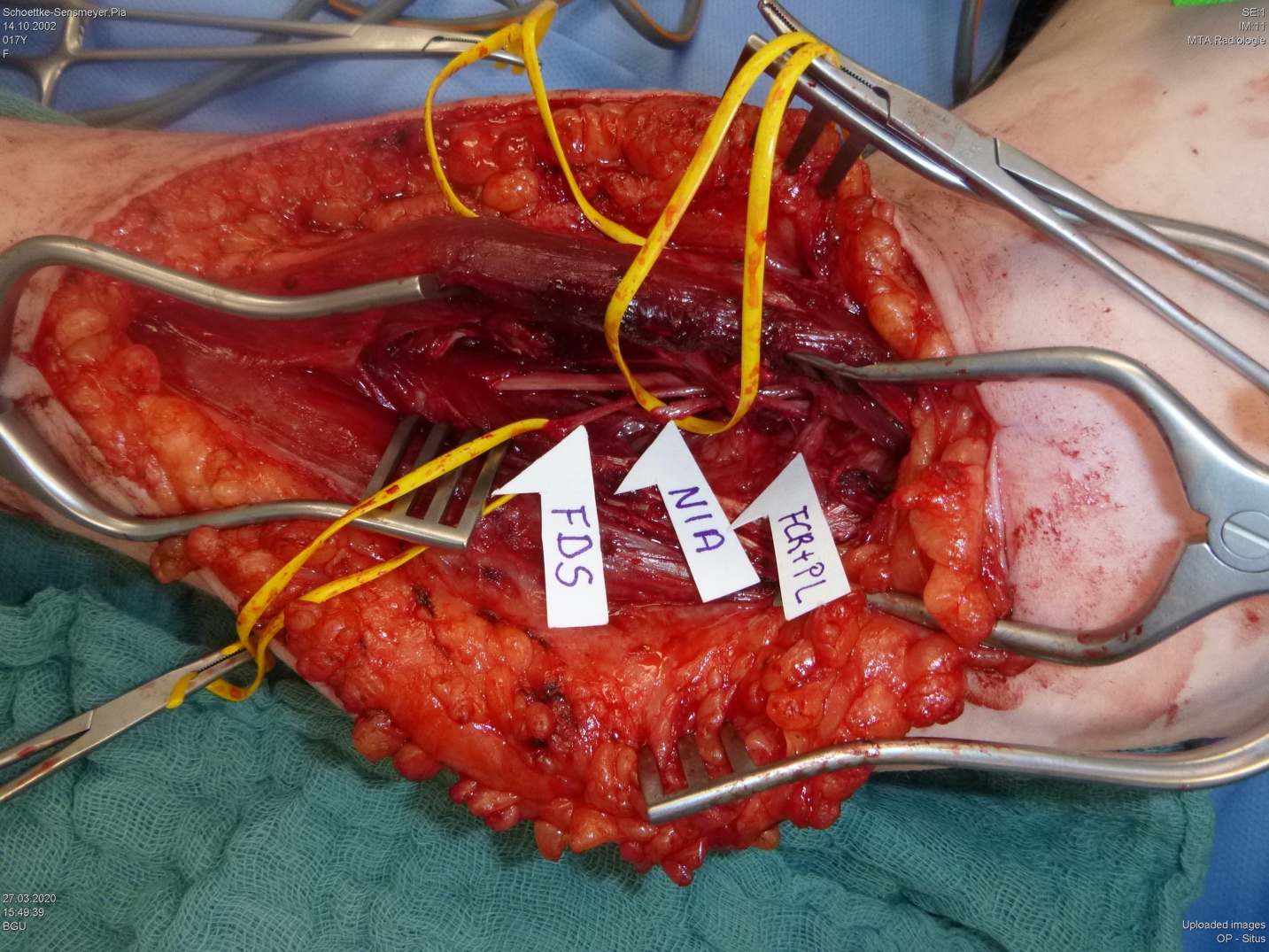


Darstellung der einzelnen Nervenabgänge des N. medianus zur Vorbereitung der hyperselektiven Neurektomie (HSN)


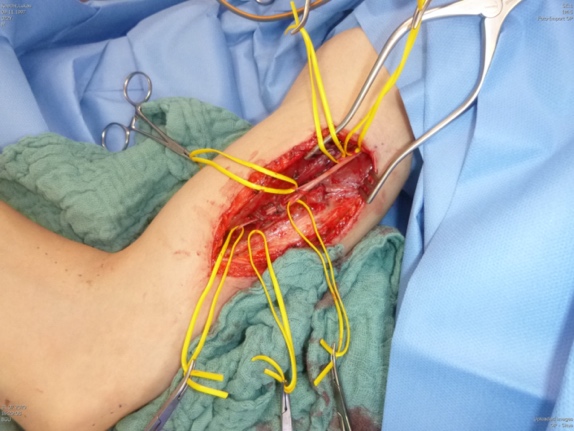


Darstellung der einzelnen Nervenabgänge des N. musculocutaneus zur Vorbereitung der hyperselektiven Neurektomie (HSN)

Die gesunden Probanden werden aus dem Mitarbeiterkreis rekrutiert. Jeder Proband wird zweimal mit einer Woche Abstand untersucht.

Dabei werden folgende Daten erhoben:

- Passive Bewegungsausmaße der Gelenke der oberen Extremität
- Aktive Bewegungsausmaße der Gelenke der oberen Extremität
- Muskelkraft (nach Janda) der Muskelgruppen der oberen Extremität
- Aktive Bewegungsausmaße der Gelenke der oberen Extremität im Rahmen der 3D-Bewegungsanalyse bei gerichteten Gelenkbewegungen sowie Alltagsbewegungen (Hand zu Mund, Hand zu Kopf, Hand zu kontralateraler Schulter) und Greifbewegungen (auf Schulterhöhe vorwärts, nach oben (Augenhöhe), auf Schulterhöhe seitwärts, Ball greifen, horizontaler Zylinder greifen, vertikaler Zylinder greifen)
- Oberflächen-EMG der Handgelenkbeuger und -strecker sowie der Ellenbogenbeuger und -strecker bei gerichteten Gelenkbewegungen sowie Alltagsbewegungen (Hand zu Mund, Hand zu Kopf, Hand zu kontralateraler Schulter) und Greifbewegungen (auf Schulterhöhe vorwärts, nach oben (Augenhöhe), auf Schulterhöhe seitwärts, Ball greifen, horizontaler Zylinder greifen, vertikaler Zylinder greifen) sowie der instrumentierten Spastik-Prüfung

Im Rahmen einer Reliabilitäts-Studie werden 20 der Patienten zusätzlich studienbedingt zur Erfassung der Test-Retest-Reliabilität 1 Tag präoperativ (während des stationären Aufenthaltes) untersucht.

Es werden die gleichen Daten, wie im Rahmen der klinischen Routine erhoben:

- Ausmaß der Spastik der betroffenen Muskelgruppen auf der modifizierten Ashworth Skala, auf der Tardieu-Skala sowie mittels instrumentierter Spastik-Prüfung (Oberflächen-EMG + Bewegungsanalyse)
- Passive Bewegungsausmaße der betroffenen Gelenke der oberen Extremität
- Aktive Bewegungsausmaße der betroffenen Gelenke der oberen Extremität
- Muskelkraft (nach Janda) der betroffenen Muskelgruppen der oberen Extremität
- Aktive Bewegungsausmaße der betroffenen Gelenke der oberen Extremität im Rahmen der 3D-Bewegungsanalyse bei gerichteten Gelenkbewegungen sowie Alltagsbewegungen (Hand zu Mund, Hand zu Kopf, Hand zu kontralateraler Schulter) und Greifbewegungen (auf Schulterhöhe vorwärts, nach oben (Augenhöhe), auf Schulterhöhe seitwärts, Ball greifen, horizontaler Zylinder greifen, vertikaler Zylinder greifen)

1. Nutzen-Risiko-Abwägung

Da im Rahmen dieser Studie nur die Dokumentation und Auswertung von in der klinischen Routine erhobenen Daten prospektiv erfolgt, bestehen keine Risiken.

Die gewonnenen Erkenntnisse sollen durch ein besseres Verständnis der postoperativen Veränderungen künftig helfen die postoperativen Ergebnisse weiter zu verbessern.

Sollten im Rahmen der Auswertung Möglichkeiten zur weiteren Verbesserung der Funktion der oberen Extremität eines einzelnen Patienten festgestellt werden, so wird im Einzelfall eine Entschlüsselung der Pseudonymisierung erfolgen und wir werden den Patienten / die Patientin bzw. die Eltern / Erziehungsberechtigten von diesen Möglichkeiten schriftlich in Kenntnis setzen.

Aus ärztlicher Sicht bestehen keine Bedenken hinsichtlich der Vertretbarkeit.

Für die Probanden besteht kein Risiko. Bei der Bewegungsanalyse werden lediglich infrarotlichtreflektierende Marker sowie Oberflächen-EMG Sensoren aufgeklebt und die Probanden bewegen die Arme.

Für die Patienten, die an der Reliabilitätsstudie teilnehmen besteht kein Risiko. Bei der Bewegungsanalyse werden lediglich infrarotlichtreflektierende Marker sowie Oberflächen-EMG Sensoren aufgeklebt und die Patienten bewegen die Arme.

1. Biometrie
   - Hypothesen-beweisende Fragestellung:

Hypothesen:

1. Die Spastik-Prüfung mittels Oberflächen-EMG und Bewegungsanalyse ist der modifizierten Ashworth-Skala und Tardieu-Skala überlegen
2. Die Therapie mittels hyperselektiver Neurotomie führt zu einer Schmerzreduktion von präoperativ zu 6 Monate postoperativ.
3. Die Schmerzreduktion nach hyperselektiver Neurotomie bleibt im Langzeitverlauf (3 Jahre, 5 Jahre, 10 Jahre postoperativ) erhalten.
4. Das aktive Bewegungsausmaß nimmt nach kombiniertem Sehnen-/ Nerveneingriff zur Therapie des spastischen Krankheitsbildes der oberen Extremität im Bereich der angrenzenden Gelenke von präoperativ zu 6 Monate postoperativ zu.
5. Das aktive Bewegungsausmaß nimmt nach kombiniertem Sehnen-/ Nerveneingriff zur Therapie des spastischen Krankheitsbildes der oberen Extremität im Bereich der angrenzenden Gelenke von 6 Monate postoperativ zu den folgenden Untersuchungszeiträumen weiter zu.
6. Das passive Bewegungsausmaß nimmt nach kombiniertem Sehnen-/ Nerveneingriff zur Therapie des spastischen Krankheitsbildes der oberen Extremität im Bereich der angrenzenden Gelenke von präoperativ zu 6 Monate postoperativ zu.
7. Das passive Bewegungsausmaß nimmt nach kombiniertem Sehnen-/ Nerveneingriff zur Therapie des spastischen Krankheitsbildes der oberen Extremität im Bereich der angrenzenden Gelenke von 6 Monate postoperativ zu den folgenden Untersuchungszeiträumen weiter zu.
8. Die Spastik sowie der Tonus der therapierten Muskulatur nehmen nach kombiniertem Sehnen-/ Nerveneingriffen zur Therapie des spastischen Krankheitsbildes der oberen Extremität von präoperativ zu 6 Monate postoperativ ab.
9. Die Spastik sowie der Tonus der therapierten Muskulatur bleiben ab 6 Monaten nach kombiniertem Sehnen-/ Nerveneingriffen zur Therapie des spastischen Krankheitsbildes der oberen Extremität konstant.
10. Die Muskelkraft im Bereich der operierten Muskulatur nimmt von prä- zu 6 Monate postoperativ nicht ab.
11. Die Muskelkraft im Bereich der operierten Muskulatur nimmt ab 6 Monaten postoperativ im Langzeitverlauf zu.
12. Bei Patienten mit ICP bleibt der der MACS-Score ab 6 Monate postoperativ konstant.
13. Es gibt in allen Ergebnisparametern präoperativ statistisch relevante Abweichungen von den gesunden Probanden, die postoperativ abnehmen.
14. Die Test-Retest Reliabilität der instrumentierten Spastik-Testung ist bei Probanden sehr gut und bei Patienten akzeptabel.
15. Die Test-Retest Reliabilität der Bewegungsausmaße und Alltagsbewegungen ist bei Probanden sehr gut und bei Patienten gut.

Größe des geplanten Stichprobenumfangs: prospektive Aufnahme aller Patienten mit spastischem Krankheitsbild an der oberen Extremität, die aufgrund dieser Diagnose in der BG Unfallklinik Ludwigshafen eine operative Therapie erhalten in die Datenbank.

10 Probanden

Einschluss von 20 Patienten in die Reliabilitätsstudie

1. Datenmanagement und Datenschutz

Die Teilnahme der Studienteilnehmer an der Studie ist freiwillig. Die Zustimmung des Patienten kann jederzeit, ohne Angaben von Gründen und ohne Nachteil für die weitere medizinische Versorgung zurückgezogen werden. Die Namen der Studienteilnehmer und alle anderen vertraulichen Informationen unterliegen der ärztlichen Schweigepflicht und den Bestimmungen der Datenschutzgrundverordnung (DSGVO) und des Bundesdatenschutzgesetzes (BDSG). Dritte erhalten keinen Einblick in Originalunterlagen.

Die für die Studie wichtigen Daten werden in pseudonymisierter Form in einer Passwort geschützten Datenbank des Motoriklabors der BG Unfallklinik Ludwigshafen gespeichert und ausgewertet. Die Daten sind gegen unbefugten Zugriff gesichert, nur die Studienleiter haben Zugriff auf die Daten. Eine Entschlüsselung erfolgt nur, wenn sich aufgrund der untersuchten Daten eine besondere (therapeutische) Konsequenz oder die Notwendigkeit zum Studienausschluss ergäbe. Die Daten werden bis 10 Jahre nach Abschluss der Studie gespeichert und dann gelöscht.

Bei Rücktritt von der Studie / Widerruf der Einwilligung kann auf Wunsch bereits gewonnenes Datenmaterial vernichtet werden. Die Studienteilnehmer können sich beim Ausscheiden aus der Studie entscheiden, ob Sie mit der Auswertung des Materials bzw. Ihrer Studiendaten einverstanden sind oder nicht und diese Entscheidung jederzeit hinsichtlich der Datenlöschung revidieren.
