# Supplementary material for: An instrumented approach to quantify wrist and finger flexor spasticity: A study protocol
Source: PLoS One. 2025 Jul 31;20(7):e0328528. doi: 10.1371/journal.pone.0328528 (PMC12312980; doi:10.1371/journal.pone.0328528)
Supplement: S3 File — (DOCX) [file pone.0328528.s003.docx]

**Study protocol**

**Project title**

Prospective objective assessment of postoperative results on the upper extremity in patients with spasticity with regard to pain, spasticity and range of motion

**Director of Studies:**

PD Dr. med. Mirjam Thielen and
Ursula Trinler, PhD
BG Klinik Ludwigshafen
(BG Kliniken Ludwigshafen und Tübingen gGmbH)
Ludwig-Guttmann-Str. 13
67071 Ludwigshafen

**Other scientists involved in teh study:**

apl. Prof. Dr. med. Leila Harhaus

Anna Pennekamp

Julia Glaser

**Participating institutions:**

BG Klinik Ludwigshafen
Ludwig-Guttmann-Str. 13
67071 Ludwigshafen

**Registration in a publicly accessible study register:**

---

**Date / Version:**

11.08.2022 / Version 4

**Summary of the project**

Spasticity, a speed-dependent increase in the resistance of muscles to stretching, is one of the leading causes of impaired motor control in the upper extremities—particularly in patients with infantile cerebral palsy, cerebral hemorrhage, stroke, and spastic incomplete tetraparesis due to high spinal cord syndrome. If left untreated, spasticity leads to muscle and later joint contractures.

Once conservative treatment options have been exhausted, and if the patient is suffering and wishes to proceed, surgical therapy can be performed. This usually consists of a combination of muscle/tendon lengthening procedures and nerve interventions. The nerve procedures are so-called hyperselective neurotomies (HSN), in which two-thirds of the nerve fibers innervating a specific muscle are selectively severed to eliminate the spasticity of that muscle without loss of strength.

The aim of this study is now, for the first time, to prospectively record all patients with a spastic condition of the upper extremity who are surgically treated at the BG Unfallklinik Ludwigshafen based on this diagnosis, in a database, and—through documentation and evaluation of objective examination results—obtain prospective short-, medium-, and long-term outcomes of the various surgical treatment methods.

1. **Scientific Background**

Spasticity is defined as a velocity-dependent increase in the resistance of muscles to passive stretching [1], which is accompanied by an increased muscle tone [2]. Spasticity is one of the leading causes of impaired motor control and, consequently, significant functional limitations in the upper extremities—particularly in patients with bilateral and unilateral spastic infantile cerebral palsy, patients after cerebral hemorrhage, patients after stroke, and patients with spastic tetraparesis in the context of high spinal cord injury [3-5]. The most common patterns in the upper extremity include shoulder adduction, internal shoulder rotation, elbow flexion, forearm pronation, wrist flexion and ulnar deviation, thumb-in-palm deformity, clenched fist, and swan-neck deformities of the fingers associated with the intrinsic-plus syndrome. If left untreated, spasticity leads to muscle and subsequently joint contractures, resulting in progressive functional loss of the upper extremity and problems with care [3, 6, 7]

Standardized assessment of these patients includes strength measurement, assessment of muscle tone and the extent of spasticity (using the Modified Ashworth Scale [8] nd the Modified Tardieu Scale [9, 10]) as well as functional testing including video recordings [6, 11, 12]. The method of 3D motion analysis now allows us to objectively assess ranges of motion in everyday movements of the upper extremities [13-16]. Instrumented spasticity testing using surface electromyography (EMG) and motion analysis is superior to both the Ashworth Scale [8] and the Tardieu Scale[9, 10] [17-20].

Conservative treatment options include physical therapy, occupational therapy, and splinting—possibly in combination with tone-inhibiting oral medications or intrathecal administration of Baclofen®. Once conservative therapies have been exhausted, and if the patient experiences significant burden and wishes treatment, surgical therapy can be considered. Surgical treatment usually consists of a combination of tendon and nerve procedures. Through intramuscular lengthening (fractional lengthening), release of the muscle aponeurosis, tendon lengthening, or if necessary, tenotomies, the overactivity of the spastic muscles is reduced and, ideally, full range of motion of adjacent joints is restored [21-40]. If this is not successful, additional arthrolysis or even arthrodesis may need to be considered [41, 42]. A second pillar of surgical therapy is hyperselective neurotomy (HSN), in which two-thirds of the nerve fibers innervating a specific muscle are severed to eliminate the spasticity of that muscle without loss of strength [3, 43-48].

Although there is general consensus among leading experts in the field of spastic hand surgery regarding the above-mentioned diagnostic and treatment procedures, to our knowledge, no study has yet prospectively and objectively (using 3D motion analysis) assessed the treatment success after surgical intervention of the upper extremity for various spastic conditions in the short, medium, and long term.

Literature:

1. Lance, J.W., *What is spasticity?* Lancet, 1990. **335**(8689): p. 606.

2. Sanger, T.D., et al., *Classification and definition of disorders causing hypertonia in childhood.* Pediatrics, 2003. **111**(1): p. e89-97.

3. Mikalef, P. and D. Power, *The role of neurectomy in the management of spasticity of the upper limb.* EFORT Open Rev, 2017. **2**(11): p. 469-473.

4. Tranchida, G.V. and A. Van Heest, *Preferred options and evidence for upper limb surgery for spasticity in cerebral palsy, stroke, and brain injury.* J Hand Surg Eur Vol, 2020. **45**(1): p. 34-42.

5. Angulo-Parker, F.J. and J.M. Adkinson, *Common Etiologies of Upper Extremity Spasticity.* Hand Clin, 2018. **34**(4): p. 437-443.

6. Leclercq, C., *General assessment of the upper limb.* Hand Clin, 2003. **19**(4): p. 557-64.

7. Rhee, P.C., *Surgical Management of the Spastic Forearm, Wrist, and Hand: Evidence-Based Treatment Recommendations: A Critical Analysis Review.* JBJS Rev, 2019. **7**(7): p. e5.

8. Bohannon, R.W. and M.B. Smith, *Interrater reliability of a modified Ashworth scale of muscle spasticity.* Phys Ther, 1987. **67**(2): p. 206-7.

9. Mackey, A.H., et al., *Intraobserver reliability of the modified Tardieu scale in the upper limb of children with hemiplegia.* Dev Med Child Neurol, 2004. **46**(4): p. 267-72.

10. Mehrholz, J., et al., *Reliability of the Modified Tardieu Scale and the Modified Ashworth Scale in adult patients with severe brain injury: a comparison study.* Clin Rehabil, 2005. **19**(7): p. 751-9.

11. Waters, P.M., et al., *Interobserver and intraobserver reliability of therapist-assisted videotaped evaluations of upper-limb hemiplegia.* J Hand Surg Am, 2004. **29**(2): p. 328-34.

12. Carlson, M.G., et al., *Impact of video review on surgical procedure determination for patients with cerebral palsy.* J Hand Surg Am, 2009. **34**(7): p. 1225-31.

13. Jaspers, E., et al., *Review of quantitative measurements of upper limb movements in hemiplegic cerebral palsy.* Gait Posture, 2009. **30**(4): p. 395-404.

14. Jaspers, E., et al., *The reliability of upper limb kinematics in children with hemiplegic cerebral palsy.* Gait Posture, 2011. **33**(4): p. 568-75.

15. Jaspers, E., et al., *Upper limb kinematics: development and reliability of a clinical protocol for children.* Gait Posture, 2011. **33**(2): p. 279-85.

16. Bar-On, L., et al., *A clinical measurement to quantify spasticity in children with cerebral palsy by integration of multidimensional signals.* Gait Posture, 2013. **38**(1): p. 141-7.

17. Bar-On, L., et al., *Manually controlled instrumented spasticity assessments: a systematic review of psychometric properties.* Dev Med Child Neurol, 2014. **56**(10): p. 932-50.

18. Bar-On, L., et al., *Is an instrumented spasticity assessment an improvement over clinical spasticity scales in assessing and predicting the response to integrated botulinum toxin type a treatment in children with cerebral palsy?* Arch Phys Med Rehabil, 2014. **95**(3): p. 515-23.

19. McGibbon, C.A., et al., *Elbow spasticity during passive stretch-reflex: clinical evaluation using a wearable sensor system.* J Neuroeng Rehabil, 2013. **10**(1): p. 61.

20. Keenan, M.A., T.T. Haider, and L.R. Stone, *Dynamic electromyography to assess elbow spasticity.* J Hand Surg Am, 1990. **15**(4): p. 607-14.

21. Tafti, M.A., S.C. Cramer, and R. Gupta, *Orthopaedic management of the upper extremity of stroke patients.* J Am Acad Orthop Surg, 2008. **16**(8): p. 462-70.

22. Namdari, S., et al., *Shoulder tenotomies to improve passive motion and relieve pain in patients with spastic hemiplegia after upper motor neuron injury.* J Shoulder Elbow Surg, 2011. **20**(5): p. 802-6.

23. Namdari, S., et al., *Outcomes of tendon fractional lengthenings to improve shoulder function in patients with spastic hemiparesis.* J Shoulder Elbow Surg, 2012. **21**(5): p. 691-8.

24. Carlson, M.G., et al., *Early results of surgical intervention for elbow deformity in cerebral palsy based on degree of contracture.* J Hand Surg Am, 2012. **37**(8): p. 1665-71.

25. Dy, C.J., et al., *Long-term results following surgical treatment of elbow deformity in patients with cerebral palsy.* J Hand Surg Am, 2013. **38**(12): p. 2432-6.

26. Anakwenze, O.A., et al., *Myotendinous lengthening of the elbow flexor muscles to improve active motion in patients with elbow spasticity following brain injury.* J Shoulder Elbow Surg, 2013. **22**(3): p. 318-22.

27. Keenan, M.A., et al., *Results of fractional lengthening of the finger flexors in adults with upper extremity spasticity.* J Hand Surg Am, 1987. **12**(4): p. 575-81.

28. Van Heest, A.E., et al., *The supination effect of tendon transfer of the flexor carpi ulnaris to the extensor carpi radialis brevis or longus: a cadaveric study.* J Hand Surg Am, 1999. **24**(5): p. 1091-6.

29. Van Heest, A.E., J.H. House, and C. Cariello, *Upper extremity surgical treatment of cerebral palsy.* J Hand Surg Am, 1999. **24**(2): p. 323-30.

30. Van Heest, A.E., et al., *Tendon transfer surgery in upper-extremity cerebral palsy is more effective than botulinum toxin injections or regular, ongoing therapy.* J Bone Joint Surg Am, 2015. **97**(7): p. 529-36.

31. Van Heest, A., et al., *Follow-up motion laboratory analysis for patients with spastic hemiplegia due to cerebral palsy: analysis of the flexor carpi ulnaris firing pattern before and after tendon transfer surgery.* J Hand Surg Am, 2010. **35**(2): p. 284-90.

32. Thevenin-Lemoine, C., et al., *Flexor origin slide for contracture of spastic finger flexor muscles: a retrospective study.* J Bone Joint Surg Am, 2013. **95**(5): p. 446-53.

33. Inglis, A.E. and W. Cooper, *Release of the flexor-pronator origin for flexion deformities of the hand and wrist in spastic paralysis. A study of eighteen cases.* J Bone Joint Surg Am, 1966. **48**(5): p. 847-57.

34. Braun, R.M., V. Mooney, and V.L. Nickel, *Flexor-origin release for pronation-flexion deformity of the forearm and hand in the stroke patient. An evaluation of the early results in eighteen patients.* J Bone Joint Surg Am, 1970. **52**(5): p. 907-20.

35. El-Said, N.S., *Selective release of the flexor origin with transfer of flexor carpi ulnaris in cerebral palsy.* J Bone Joint Surg Br, 2001. **83**(2): p. 259-62.

36. Peraut, E., et al., *Results and complications of superficialis-to-profundus tendon transfer in brain-damaged patients, a series of 26 patients.* Orthop Traumatol Surg Res, 2018. **104**(1): p. 121-126.

37. Pomerance, J.F. and M.A. Keenan, *Correction of severe spastic flexion contractures in the nonfunctional hand.* J Hand Surg Am, 1996. **21**(5): p. 828-33.

38. Davids, J.R., et al., *Validation of the Shriners Hospital for Children Upper Extremity Evaluation (SHUEE) for children with hemiplegic cerebral palsy.* J Bone Joint Surg Am, 2006. **88**(2): p. 326-33.

39. Davids, J.R., et al., *Surgical management of thumb deformity in children with hemiplegic-type cerebral palsy.* J Pediatr Orthop, 2009. **29**(5): p. 504-10.

40. Alewijnse, J.V., M.J. Smeulders, and M. Kreulen, *Short-term and Long-term Clinical Results of the Surgical Correction of Thumb-in-Palm Deformity in Patients With Cerebral Palsy.* J Pediatr Orthop, 2015. **35**(8): p. 825-30.

41. Van Heest, A.E. and D. Strothman, *Wrist arthrodesis in cerebral palsy.* J Hand Surg Am, 2009. **34**(7): p. 1216-24.

42. Rayan, G.M. and B.T. Young, *Arthrodesis of the spastic wrist.* J Hand Surg Am, 1999. **24**(5): p. 944-52.

43. Gras, M. and C. Leclercq, *Spasticity and hyperselective neurectomy in the upper limb.* Hand Surg Rehabil, 2017. **36**(6): p. 391-401.

44. Kwak, K.W., et al., *Surgical results of selective median neurotomy for wrist and finger spasticity.* J Korean Neurosurg Soc, 2011. **50**(2): p. 95-8.

45. Puligopu, A.K. and A.K. Purohit, *Outcome of selective motor fasciculotomy in the treatment of upper limb spasticity.* J Pediatr Neurosci, 2011. **6**(Suppl 1): p. S118-25.

46. Maarrawi, J., et al., *Long-term functional results of selective peripheral neurotomy for the treatment of spastic upper limb: prospective study in 31 patients.* J Neurosurg, 2006. **104**(2): p. 215-25.

47. Purohit, A.K., et al., *Selective musculocutaneous fasciculotomy for spastic elbow in cerebral palsy: a preliminary study.* Acta Neurochir (Wien), 1998. **140**(5): p. 473-8.

48. Brunelli, G. and F. Brunelli, *Partial selective denervation in spastic palsies (hyponeurotization).* Microsurgery, 1983. **4**(4): p. 221-4.

1. Project goals

The aim of this study is, for the first time, to prospectively record all patients with a spastic condition of the upper extremity who undergo surgery for this diagnosis at the BG Unfallklinik Ludwigshafen in a database, and to investigate the short-, medium-, and long-term outcomes of the various surgical treatment procedures through documentation and evaluation of objective examination results.

1. Target values

- Primary targets:

Measured at all time points (1 week preoperatively, 6 months postoperatively, 1 year postoperatively, 3 years postoperatively, 5 years postoperatively, 10 years postoperatively):

- Pain on the Visual Analog Scale (VAS)
- Pain questionnaire "Pain-Detect"
- Extent of spasticity of the affected muscle groups according to the Modified Ashworth Scale, the Tardieu Scale, and instrumented spasticity testing (surface EMG + motion analysis)
- Passive range of motion of the affected joints (elbow, wrist, metacarpophalangeal joints, and interphalangeal joints) of the upper extremity
- Active range of motion of the affected joints of the upper extremity
- Muscle strength (according to Janda) of the affected muscle groups of the upper extremity
- Active range of motion of the affected joints (elbow extension/flexion, wrist extension/flexion, wrist radial/ulnar deviation, forearm pronation/supination, metacarpophalangeal joint extension/flexion, and interphalangeal joint extension/flexion) of the upper extremity, assessed using 3D motion analysis during directed joint movements and everyday movements (hand to mouth, hand to head, hand to contralateral shoulder) and grasping movements (forward at shoulder height, upward at eye level, sideways at shoulder height, grasping a ball, grasping a horizontal cylinder, grasping a vertical cylinder)
- For patients diagnosed with infantile cerebral palsy: MACS Score (Manual Ability Classification System)

1. Study population

- Inclusion criteria:
  - Patients with spasticity of the upper extremity and an indication for surgery to treat this condition
- Exclusion criteria:
  - Severe intellectual disability (inability to give consent or understand/perform targeted movement tasks)
  - Non-compliance and/or inability to provide informed consent
  - Botulinum toxin injection in the affected upper extremity within the last 6 months
  - Patients unable to give consent
- Number of study participants:

If possible, all patients with a spastic condition of the upper extremity who receive surgical treatment for this diagnosis at the BG Unfallklinik Ludwigshafen should be prospectively included in the database with immediate effect.

- Recruitment measures:
  Patients are recruited during their outpatient appointment in the specialized consultation, during which the surgical indication is determined.

Control group (test subjects):
10 consenting adults without pre-existing conditions in the area of the upper extremities.

1. Methodology and Implementation

This is a monocentric study. Patient information and consent are obtained during their outpatient appointment in the specialized consultation, where the surgical indication is determined. All data are collected prospectively.

As part of treatment planning and postoperative follow-up, patients with spasticity undergo standardized instrumental movement analyses and clinical examinations, including pain assessment, at regular intervals (1 week preoperatively, 6 months postoperatively, 1 year postoperatively, 3 years postoperatively, 5 years postoperatively, 10 years postoperatively). This is conducted as part of routine clinical practice. All data required for the study evaluation are collected during these routine clinical examinations. Therefore, no additional examinations are required for study participants. Only the pseudonymization and evaluation of the routinely collected data is carried out in a database.

- - List / description of the data to be recorded

at each measurement time point: (1 week pre-op, 6 months post-op, 1 year post-op, 3 years post-op, 5 years post-op, 10 years post-op):

- Pain on the Visual Analog Scale (VAS)
- Pain questionnaire "Pain detect"
- Extent of spasticity of the affected muscle groups according to the Modified Ashworth Scale, the Tardieu Scale, and via instrumented spasticity testing (surface EMG + motion analysis)
- Passive range of motion of the affected joints of the upper extremity
- Active range of motion of the affected joints of the upper extremity
- Muscle strength (according to Janda) of the affected muscle groups of the upper extremity
- Active range of motion of the affected joints of the upper extremity, assessed via 3D motion analysis for directed joint movements and everyday movements (hand to mouth, hand to head, hand to contralateral shoulder) and grasping movements (forward at shoulder height, upward at eye level, sideways at shoulder height, grasping a ball, grasping a horizontal cylinder, grasping a vertical cylinder)
- For patients with a diagnosis of infantile cerebral palsy: MACS Score (Manual Ability Classification System)

Markerset and EMG electrodes


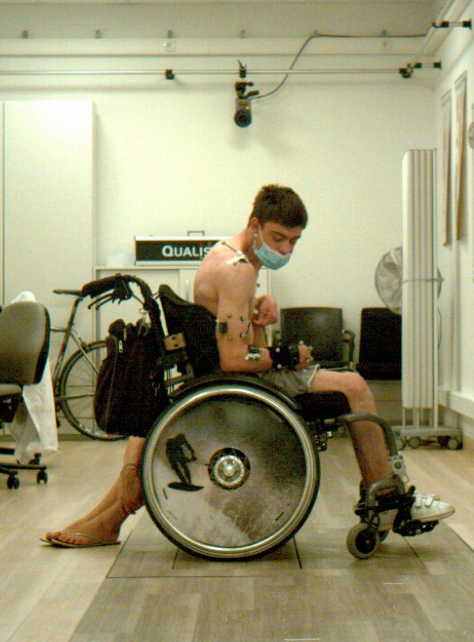

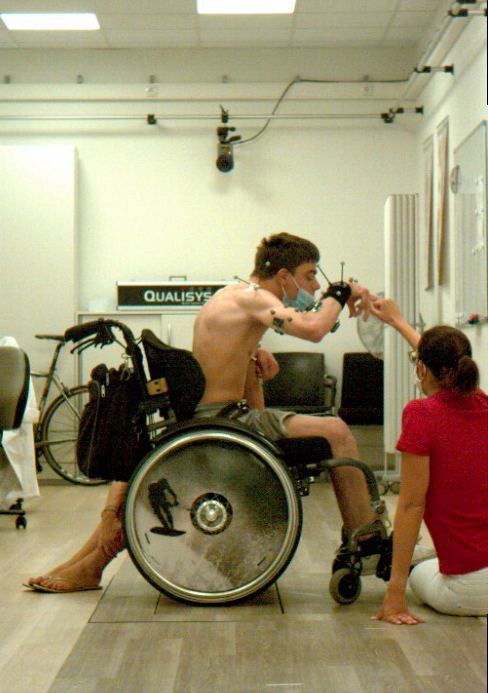

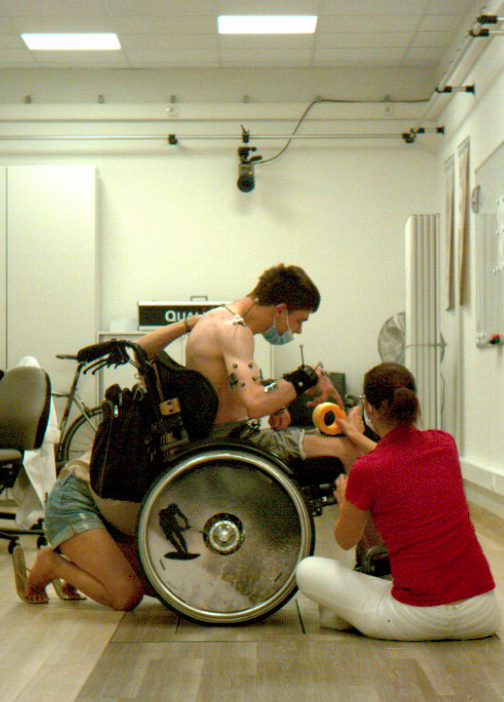


Grasping upwards (eye level)

Grasping a horizontal cylinder


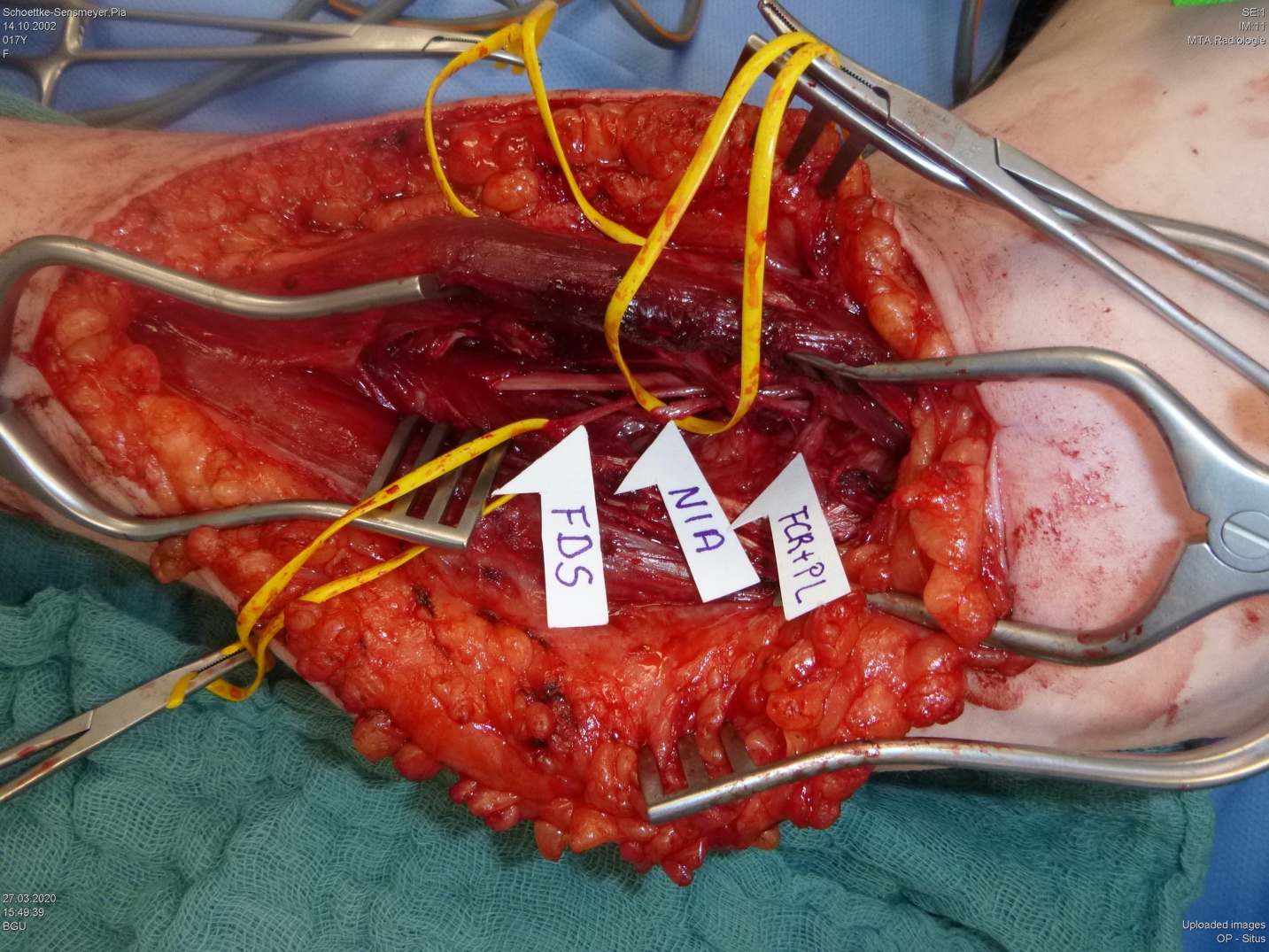


Visualization of the individual nerve branches of the meidan nerve in preparation for hyperselective neurectomy (HSN)


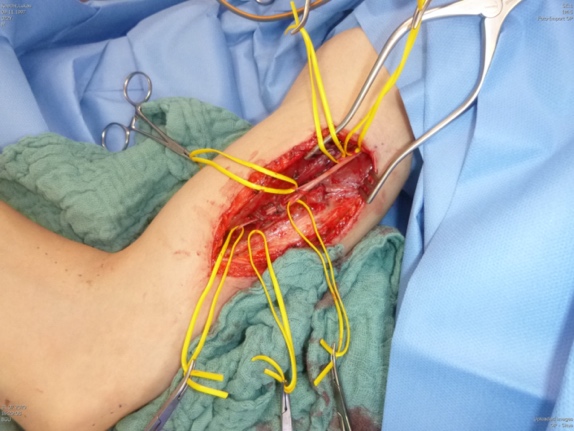


Visualization of the individual nerve branches of the musculocutaneous nerve in preparation for hyperselective neurectomy (HSN)

The healthy test subjects are recruited from among the staff. Each subject is examined twice, one week apart.

The following data is collected:

- Passive range of motion of the joints of the upper extremity
- Active range of motion of the joints of the upper extremity
- Muscle strength (according to Janda) of the muscle groups of the upper extremity
- Active range of motion of the joints of the upper extremity as part of 3D motion analysis for directed joint movements and everyday movements (hand to mouth, hand to head, hand to contralateral shoulder) and grasping movements (forward at shoulder height, upward at eye level, sideways at shoulder height, grasping a ball, grasping a horizontal cylinder, grasping a vertical cylinder)
- Surface EMG of the wrist flexors and extensors as well as the elbow flexors and extensors during directed joint movements and everyday movements (hand to mouth, hand to head, hand to contralateral shoulder) and grasping movements (forward at shoulder height, upward at eye level, sideways at shoulder height, grasping a ball, grasping a horizontal cylinder, grasping a vertical cylinder) as well as during instrumented spasticity testing

As part of a reliability study, 20 of the patients are additionally examined 1 day preoperatively (during their inpatient stay) to assess test-retest reliability.

The same data are collected as during routine clinical practice:

- Extent of spasticity of the affected muscle groups using the Modified Ashworth Scale, the Tardieu Scale, and instrumented spasticity testing (surface EMG + motion analysis)
- Passive range of motion of the affected joints of the upper extremity
- Active range of motion of the affected joints of the upper extremity
- Muscle strength (according to Janda) of the muscle groups of the upper extremity
- Active range of motion of the joints of the upper extremity as part of 3D motion analysis for directed joint movements and everyday movements (hand to mouth, hand to head, hand to contralateral shoulder) and grasping movements (forward at shoulder height, upward at eye level, sideways at shoulder height, grasping a ball, grasping a horizontal cylinder, grasping a vertical cylinder)

1. Risk-benefit assessment

Since this study solely involves the **prospective documentation and evaluation of data collected during routine clinical care**, **there are no risks** for participants.

The insights gained should help to further improve postoperative outcomes in the future through a better understanding of postoperative changes.

If, during data analysis, opportunities for further functional improvement of an individual patient’s upper limb are identified, **pseudonymization will be lifted on a case-by-case basis**, and the patient or their parents/legal guardians will be **informed in writing** of these possibilities.

From a medical standpoint, there are no concerns regarding the justifiability of the study.

There is **no risk for the test subjects**. During the motion analysis, only **infrared-reflective markers and surface EMG sensors** are applied, and the subjects move their arms.

There is **no risk for the patients participating in the reliability study**. They also undergo motion analysis with only infrared-reflective markers and surface EMG sensors applied while moving their arms.

1. Biometrics
   - Hypothetis-proving question:

Hypotheses:

1. Spasticity testing using surface EMG and motion analysis is superior to the Modified Ashworth Scale and the Tardieu Scale.
2. Treatment with hyperselective neurotomy leads to a reduction in pain from preoperative to 6 months postoperative.
3. The pain reduction after hyperselective neurotomy is maintained in the long term (3 years, 5 years, 10 years postoperative).
4. The active range of motion increases from preoperative to 6 months postoperative after combined tendon/nerve surgery for the treatment of spasticity of the upper extremity in the adjacent joints.
5. The active range of motion continues to increase from 6 months postoperative to subsequent follow-up periods after combined tendon/nerve surgery for the treatment of spasticity of the upper extremity in the adjacent joints.
6. The passive range of motion increases from preoperative to 6 months postoperative after combined tendon/nerve surgery for the treatment of spasticity of the upper extremity in the adjacent joints.
7. The passive range of motion continues to increase from 6 months postoperative to subsequent follow-up periods after combined tendon/nerve surgery for the treatment of spasticity of the upper extremity in the adjacent joints.
8. Spasticity and muscle tone of the treated muscles decrease from preoperative to 6 months postoperative after combined tendon/nerve interventions for the treatment of upper limb spasticity.
9. Spasticity and muscle tone of the treated muscles remain constant starting from 6 months after combined tendon/nerve interventions for the treatment of upper limb spasticity.
10. Muscle strength in the area of the operated muscles does not decrease from preoperative to 6 months postoperative.
11. Muscle strength in the area of the operated muscles increases in the long term starting from 6 months postoperative.
12. In patients with ICP (infantile cerebral palsy), the MACS score remains constant from 6 months postoperative onward.
13. There are statistically significant preoperative deviations from healthy subjects in all outcome parameters, which decrease postoperatively.
14. The test-retest reliability of instrumented spasticity testing is very good in healthy subjects and acceptable in patients.
15. The test-retest reliability of range of motion and everyday movements is very good in healthy subjects and good in patients.

Planned sample size: Prospective inclusion in the database of all patients with a spastic condition of the upper extremity who receive surgical treatment for this diagnosis at the BG Unfallklinik Ludwigshafen

10 test subjects (healthy controls)

Inclusion of 20 patients in the reliability study

1. Data management and data protection

Participation in the study is voluntary. The patient's consent may be withdrawn at any time without providing reasons and without any disadvantage to further medical care. The names of the study participants and all other confidential information are subject to medical confidentiality as well as the provisions of the General Data Protection Regulation (DSGVO) and the Federal Data Protection Act (BDSG). Third parties will not have access to original documents.

The data relevant to the study are stored and evaluated in pseudonymized form in a password-protected database of the motor skills laboratory at BG Unfallklinik Ludwigshafen. The data are protected against unauthorized access, and only the study directors have access. Decryption will only occur if the analyzed data result in a specific (therapeutic) consequence or if study exclusion becomes necessary. The data will be stored for up to 10 years after completion of the study and will then be deleted.

In the event of withdrawal from the study or revocation of consent, any data already collected may be destroyed upon request. When withdrawing, participants may decide whether or not they agree to the analysis of their material or study data—and they may revise this decision at any time regarding data deletion.
